# Establishing the Structural Validity, Language‐Based Performance, and Convergent Validity of the Caregiver Strain Questionnaire‐Short Form 11 Within a Latinx Sample

**DOI:** 10.1111/famp.70213

**Published:** 2026-11-01

**Authors:** Alejandro L. Vázquez, Cynthia M. Navarro Flores, Melanie M. Domenech Rodríguez, Mary L. Held, Amanda Venta, Oswaldo Moreno, Thania Galvan, Oscar F. Perez Rojas, Germán A. Cadenas

**Affiliations:** ^1^ University of Tennessee Knoxville Tennessee USA; ^2^ Utah State University Logan Utah USA; ^3^ University of Houston Houston Texas USA; ^4^ Arizona State University Tempe Arizona USA; ^5^ University of Georgia Athens Georgia USA; ^6^ Yale University School of Medicine New Haven Connecticut USA; ^7^ Rutgers University New Brunswick New Jersey USA

**Keywords:** caregiver strain, Latinx families, Latinx youth, measurement, Spanish

## Abstract

Caregiver strain or stress resulting from caring for a child with emotional/behavioral problems—can negatively impact the psychological health of caregivers and may underly decisions to seek mental health services for their youth. The Caregiver Strain Questionnaire‐Short Form 11 (CGSQ‐SF11) was developed to assess caregiver strain, but a Spanish language form has yet to be evaluated, limiting its utility with Latinx families. This study evaluated the CGSQ‐SF11 as a tool for assessing caregiver strain among Latinx caregivers. The sample included 511 Latinx caregivers of youths aged 6–18 who self‐selected into English (*n* = 275) or Spanish (*n* = 236) questionnaires. In general, caregivers reported moderate levels of caregiver strain, and caregivers who completed the Spanish survey reported significantly lower youth mental health problem severity and caregiver strain relative to those in the English survey. Confirmatory factor analyses suggest that a three‐factor structure fit the data better (i.e., objective, subjective internalized, and subjective externalized strain) than a global factor. However, a bifactor model suggested that the variance in the total score was best represented by a unidimensional structure. The CGSQ‐SF11 achieved metric invariance, which suggests that the latent variable and strength of factor loadings for the global scale were similar across language forms. Finally, we provide evidence of convergent validity through associations between the CGSQ‐SF11 global scale and measures of youth internalizing and externalizing problems. Our findings support the use of CGSQ‐SF11 English and Spanish language forms with Latinx caregivers.

## 1 Introduction

Latinx families' interpretations and responses to their youths' emotional and/or behavioral problems are influenced by their perceptions of the severity of the problem (Galvan and Gudiño 2020). For instance, Latinx caregivers, or family member who regularly care for a child may be more likely to seek services for some types of youth problems (e.g., internalizing problems such as anxiety and depression) and not others (e.g., externalizing problems such as aggression, oppositionality, rule‐breaking; Galvan and Gudiño 2021). Latinx caregivers may also be more likely to normalize clinically elevated youth emotional and/or behavioral problems, leading to delays in recognizing the need for and seeking mental health services (Vázquez et al. 2022). *Caregiver strain*—the stress resulting from caring for a child with emotional and/or behavioral problems—has been identified as a key factor for understanding how caregivers recognize youth mental health problems and attempt to cope with related psychological distress by seeking mental health services for their youth (Brannan et al. 2003; Galvan and Gudiño 2020). However, a widely used measure of caregiver strain (e.g., Caregiver Strain Questionnaire [CGSQ]), has been primarily developed and evaluated on predominantly English‐speaking, non‐Latinx White populations thus limiting the utility of this scale in Latinx populations (Brannan et al. 1997). This measurement gap limits researchers and clinical practitioners' ability to assess perceptual factors contributing to disparities in mental health and psychological service access among Latinx families (Domenech Rodríguez et al. 2024). The present study sought to improve access through a linguistically accessible psychological measures to assess caregiver strain among Latinx caregivers.

A conceptual model developed by Brannan and Heflinger (2001) describes the process by which caregiver strain emerges in responses to youth emotional and behavioral problems. Within this model, youth symptoms and life stressors pile up across time contributing to the development of caregiver strain and related psychological distress. This process is mediated by family resources (e.g., time and material resources; access to social supports and mental health services) and perceptions of the youth mental health problems and other life stressors (e.g., interpretations of stressors; perceived health risk for the child) that can buffer against the development of caregiver strain and related psychological distress. Further, caregiver strain alerts and motivates caregivers to seek mental health services for their child as an active coping strategy to reduce caregiver strain and associated psychological distress (Brannan et al. 2003). Thus, caregiver strain is an important construct in health disparities research as it can influence the psychological health of caregivers and may underlie decisions to seek mental health services on behalf of their children.

The CGSQ, developed by Brannan et al. (1997), is a widely used measure designed to assess the impact of caring for a youth with emotional and/or behavioral problems. This measure is distinct from measures that assess parenting stress more broadly (e.g., Parenting Stress Index–Short Form [PSI‐SF]; Reitman et al. 2002). The CGSQ includes a global caregiver strain scale and three subscales relating to objective, subjective internalized, and subjective externalized strain. The objective strain subscale evaluates tangible disruptions caused by caring for a child with emotional and/or behavioral problems, such as financial strain, missed work, and family disruptions (Brannan et al. 1997). The subjective internalized strain subscale reflects the psychological toll of caregiving, such as sadness, worry, and exhaustion (Brannan et al. 1997). Finally, the subjective externalized strain subscale measures caregivers' negative feelings toward the child, including anger, resentment, or embarrassment (Brannan et al. 1997). However, the cultural and linguistic appropriateness of the CGSQ for Latinx populations has not been established, potentially neglecting to measure important sources of caregiver strain (Domenech Rodríguez et al. 2024; Galvan and Gudiño 2020).

Research suggests that caregiver strain is a common experience among Latinx caregivers of youth with emotional and/or behavioral problems (Domenech Rodríguez et al. 2024). Latinx caregivers experience caregiver strain from both youth internalizing and externalizing problems (Galvan and Gudiño 2020). Prior research has identified caregiver strain as an important correlate of caregivers' perceived need for and utilization of youth mental health services (Vázquez, Chou, et al. 2021), which suggests that increases in caregiver strain can alert caregivers to the need to seek formal mental health services for their youth (Brannan et al. 2003; Vázquez, Culianos, et al. 2024). Furthermore, caring for a child with mental health problems can be a significant burden, which contributes to an increased risk for developing psychopathology among caregivers (Brennan et al. 2021). While research has highlighted the importance of assessing caregiver strain, interpretations of the severity of youth mental health problems and associated help‐seeking behaviors vary across and within cultural groups (Brannan et al. 2003; Vázquez, Chou, et al. 2021). Studying how caregiver strain operates in Latinx communities is essential for reducing mental health service disparities among youths and supporting the psychological well‐being of caregivers (Galvan and Gudiño 2021).

The CGSQ has been criticized for the lack of diverse racial and ethnic representation in psychological evaluation studies (i.e., originally included 75% White participants; Brannan et al. 1997; Domenech Rodríguez et al. 2024). While the CGSQ is widely used in health disparities research (Galvan and Gudiño 2020, 2021), few studies have evaluated the psychometric properties of the CGSQ among Latinx caregivers. Recent research by Domenech Rodríguez et al. (2024) sought to address this gap by establishing the structure, internal, and convergent validity of 21‐item English CGSQ among Latinx caregivers. This study confirmed the three‐factor solution, established convergent validity through positive correlations with youth internalizing/externalizing problem severity and mental health service use and recommended removing a reverse coded item to improve model fit within this population (i.e., final 20‐item scale; removed item 14: “How well did you relate to your child?”). In this work, the absence of a psychometric evaluation of a Spanish CGSQ form was noted as a limitation and next step.

Another challenge limiting the utility of the CGSQ is the availability of a short form that can be efficiently administered in research and clinical settings with Latinx populations. Prior research has sought to reduce the administration burden for respondents by developing shorter versions of the CGSQ, such as the seven‐ and 11‐item short forms (Brannan et al. 2012, 2021). Both brief versions of the CGSQ exclude the reverse coded item 14 that was previously identified as not functioning correctly within a Latinx sample, which provides some indication that these measures may perform well within this population (Domenech Rodríguez et al. 2024). The CGSQ‐SF7 allows for the estimation of global strain (Cronbach's *α* = 0.89) and two subscales: objective (4 items; *α* = 0.84) and subjective strain (3 items; *α* = 0.86; Brannan et al. 2012). A recent 11‐item form was developed to allow for estimation of a global scale (*α* = 0.96) and the original three subscales: objective (4 items, *α* = 0.91), subjective internalized (4 items, *α* = 0.90), and subjective externalized strain (3 items; *α* = 0.88). While these versions improved the feasibility of administering the CGSQ, validation studies have included limited representation of Latinx caregivers (CGSQ‐SF7 29% Black, 9% all other groups; CGSQ‐SF11 12.6% Latinx caregivers; Brannan et al. 2012, 2021). There are no known examinations of a Spanish brief CGSQ form. As a result, available versions of the CGSQ may have limited clinical utility and lead to research that potentially misrepresents the experiences of Latinx caregivers due to a lack of language accessibility that contributes to more acculturated samples and/or measurement error resulting from the use of a tool that has not been validated with the target population (Domenech Rodríguez et al. 2024).

The present study was designed to address a measurement gap by conducting a psychometric evaluation of a Spanish translation of the CGSQ‐SF11 to increase language accessibility for Latinx caregivers. The study aimed to (a) determine the optimal factor structure of the CGSQ‐SF11 within a sample of English and Spanish‐speaking Latinx caregivers, (b) determine language‐based equivalence between the English and Spanish forms, and (c) explore convergent validity by examining associations between the CGSQ‐SF11 and indicators representing youth internalizing and externalizing mental health problems. Based on prior research, the CGSQ‐SF11 was expected to be best represented by the three‐factor structure, and scales were generally expected to be positively correlated with youth internalizing/externalizing problem severity (Domenech Rodríguez et al. 2024). As prior research has not examined measurement invariance of CGSQ‐SF11 language versions, analyses were exploratory in nature.

## 2 Method

### 2.1 Procedures

The present study used extant data collected as part of the second phase of the Pathways to Latinx Mental Health study, which examined youth mental health service utilization patterns and their related correlates among Latinx caregivers (Vázquez, Domenech Rodríguez, Cadenas, et al. 2024). This phase recruited 511 Latinx caregivers of youths aged 6–18 through a QuestionPro survey panel conducted between August 24, 2021, and April 20, 2022. QuestionPro survey panels are comprised of individuals interested in being contacted to participate in research opportunities. Using a survey panel ensured efficient, cost‐effective ($20.00 per participant), and safe data collection during the Coronavirus Pandemic.

QuestionPro invited potential participants via email, providing details about the survey, expected duration, and compensation. Interested caregivers completed a screener to confirm their eligibility. Inclusion criteria included (a) identifying as Latinx, (b) being a caregiver to a youth aged 6–18, (c) residing in the United States (U.S.), and (d) having a child with emotional or behavioral problems. Caregivers were asked to read a letter of information and provided informed consent to participate before continuing to the survey. Those who were eligible and consented to participate completed a 30‐min online survey. Caregivers with multiple children were instructed to report on the child that presented the most challenges to them as a parent. Caregivers self‐selected into an English or Spanish survey based on their preference. Responses were required for all items in the survey. Consistent with best practices for protecting data integrity against bots and malicious respondents, only participants who provided complete data and passed all validity checks were included and compensated for completing the survey (Kennedy et al. 2020; Lowry et al. 2016). Poor‐quality responses were defined as respondents who failed at least one validation method including inconsistent responses between logical statements, incorrect responses to directed queries, response time that was flagged by QuestionPro as being significantly below the median, and failing the reCAPTCHA (Abbey and Meloy 2017). Compensation was in the form of points that could be redeemed for rewards from QuestionPro.

Of 6439 respondents who opened the study link (English = 3007; Spanish = 3432), 1367 met the eligibility criteria. Participants that provided low‐quality data (*n* = 658; English = 499, Spanish = 159) or incomplete surveys (*n* = 193; English = 104, Spanish = 89), or did not consent to participate (*n* = 5) were excluded, consistent with best practices for survey panel research (Abbey and Meloy 2017). These procedures were reviewed and approved by the Utah State University Institutional Review Board (#12087). Analysis of the deidentified extant data was approved as nonhuman subjects research by the University of Tennessee, Knoxville Institutional Review Board.

### 2.2 Measures

#### 2.2.1 Eligibility Screening

Caregivers completed five screening questions to confirm their eligibility for the study including whether they resided within the U.S. (i.e., *yes* or *no*), what racial/ethnic group they most identified with (i.e., White, Black/African American, Latina/o/x, Asian/Asian American, Native American, Pacific Islander, other), whether they were a caregiver to a youth (i.e., two items [1] below age 19, [2] between ages of 6–18), and whether that youth had behavioral or emotional problems (i.e., *yes* or *no*).

#### 2.2.2 Sociodemographic

Caregivers were asked to report their demographic information (e.g., age, binary gender, generation in the U.S., preferred language, relation to the child, education) and that of their child (i.e., age, binary gender, health insurance status). Caregivers also reported their subjective socioeconomic status (MacArthur Scale of Subjective Social Status; Adler and Stewart 2007). Caregivers were presented with a picture of a ladder with 10 rungs. Responses ranged from selecting the top rung representing the highest socioeconomic status in their community (10) and the lowest rung representing the worse off in their community (1).

#### 2.2.3 Caregiver Strain

The CGSQ‐SF11 assesses the impact of caring for a child with emotional and/or behavioral problems (Brennan et al. 2021). The CGSQ‐SF11 is composed of a global scale and three subscales: Objective, subjective externalized, and subjective internalized strain. The CGSQ‐SF11 asks caregivers to report how much of a problem each statement was “at the present time.” Responses were: (1) *not at all*, (2) *a little*, (3) *somewhat*, (4) *quite a bit*, or (5) *very much*. The global scale was calculated by taking the average of all 11 items, with higher scores representing greater caregiver strain. Subscales were calculated as an average of items composing the objective (4‐item), subjective externalized (3‐item), and subjective internalized strain scales (4‐item). See Supporting Information for the full measure.

#### 2.2.4 Youth Mental Health Problems

The Child Behavior Checklist asks caregivers to report the frequency of their youths' emotional and behavioral problems in the last six months (CBCL; 113 items; ages 6–18; Achenbach and Rescorla 2001). Responses were (0) *not true*, (1) *sometimes true*, or (2) *often true*. Two composite *t* scores were calculated for youth internalizing (e.g., depression, anxiety) and externalizing problems (e.g., aggression, rule‐breaking). The CBCL had excellent internal consistency with the present sample (English *α* = 0.95 internalizing; *α* = 0.96 externalizing; Spanish *α* = 0.93 internalizing; *α* = 0.95 externalizing) and was similar to those published in the CBCL manual (internalizing *α* = 0.95; externalizing *α* = 0.96; Achenbach and Rescorla 2001).

#### 2.2.5 Measure Translation

A committee approach was used to translate sociodemographic items and the CGSQ‐SF11 from English to Spanish (Beaton et al. 2000). The survey was forward‐translated from English to Spanish by a research team member. Another team member then back‐translated the Spanish survey to English. A third team member then resolved discrepancies between versions and introduced changes to the final Spanish survey. The CBCL was not translated as it was already available in Spanish.

### 2.3 Participants

The final sample consisted of 511 (English survey = 275, Spanish survey = 236) Latinx caregivers of youths ages 6–18. Caregivers were roughly 39 years old on average (*M* = 38.8, SD = 6.7), predominately female (61.6%, *n* = 315), most frequently reported being first (38%, *n* = 194) or second generation in the U.S. (31.9%, *n* = 163), preferred English and Spanish equally (40.9%, *n* = 209), were the biological parent (95.3%, *n* = 487), had a post high school education (67.3%, *n* = 344), and were of moderate to high subjective economic status (*M* = 6.4, SD = 1.9). Youth were roughly 12 years old on average (*M* = 11.9, SD = 3.6), were predominately male (62%, *n* = 317), and predominately had health insurance (94.7%, *n* = 484). See Table 1 for sample characteristics by survey version.

**TABLE 1:** Sample Characteristics.

	Total	English	Spanish	*p*
	(*N* = 511)	(*n* = 275; 53.8%)	(*n* = 236; 46.2%)	
	*M* (SD)	*M* (SD)	*M* (SD)	
*Caregiver*				
Age	38.76 (6.68)	38.24 (6.83)	39.37 (6.46)	
Sex				< 0.001
Male	196 (38.4%)	127 (46.2%)	69 (29.2%)	
Female	315 (61.6%)	148 (53.8%)	167 (70.8%)	
Generation in U.S.				< 0.001
1st generation	194 (38%)	40 (14.5%)	154 (65.3%)	
2nd generation	163 (31.9%)	102 (37.1%)	61 (25.8%)	
3rd generation	65 (12.7%)	55 (20%)	10 (4.2%)	
4th or greater	89 (17.4%)	78 (28.4%)	11 (4.7%)	
Biological parent	487 (95.3%)	265 (96.4%)	222 (94.1%)	
Post‐high school education	344 (67.3%)	210 (76.4%)	134 (56.8%)	< 0.001
SES	6.41 (1.86)	6.64 (1.94)	6.13 (1.71)	< 0.001
*Child*				
Age	11.94 (3.61)	11.67 (3.74)	12.27 (3.44)	
Sex				
Male	317 (62%)	174 (63.3%)	143 (60.6%)	
Female	194 (38%)	101 (36.7%)	93 (39.4%)	
Internalizing *T* score	64.31 (13.68)	66.28 (13.90)	62.01 (13.09)	< 0.001
Externalizing *T* score	61.96 (12.85)	64.39 (13.05)	59.13 (12.03)	< 0.001

*Note:* Chi‐square tests of independence were used for dichotomous variables. Kruskal‐Wallis rank sum tests were used for continuous variables.

Abbreviations: SES, subjective economic status; U.S., United States.

### 2.4 Analytic Plan

Descriptive statistics were used to characterize the sample and responses on the CGSQ‐SF11 (i.e., chi‐square test of independence, Kruskal‐Wallis rank sum test, Kendall's Tau bivariate correlation). Confirmatory factor analysis (CFA) with the Diagonally Weighted Least Square estimator was used to confirm the factor structure of the CGSQ‐SF11 within the present sample in three steps (Kline 2023; Rodriguez et al. 2016). As we used extant data, suitability for CFA analysis of the CGSQ‐SF11 was determined based on the 10 participants per parameter estimated guideline, which would suggest a minimum necessary sample size of 110 for the global model and 140 for the three‐factor model (Brown 2015). First, a unidimensional model was fitted with all CGSQ‐SF11 items loading onto a global factor. Second, a three‐factor model was fitted with items loading onto objective, subjective internalized, and subjective externalized subscales. Third, an orthogonal bifactor model was estimated with scale items fitted onto the global and three‐factor solutions simultaneously to determine whether a multidimensional solution was supported beyond a unidimensional factor (Rodriguez et al. 2016).

Decisions regarding the optimal factor structure were based on multiple indicators (Brown 2015) applied to modeling with the full sample (including both English and Spanish versions). Evaluation of the optimal factor solution was guided by approximation to recommended cut‐offs on multiple fit indices, as they were not developed for use with ordinal data (Root Mean Square Error of Approximation [RMSEA; good < 0.06, acceptable < 0.08, poor fit > 0.10], Comparative Fit Index [CFI; ≥ 0.95 good fit], Tucker‐Lewis Index [TLI; good fit ≥ 0.95], Standardized Root Mean Square Residual [SRMR; acceptable fit ≤ 0.08]; Kline 2023). As scaled chi‐square difference tests are not recommended for ordinal estimators (Kline 2023), we focused on changes in CFI and/or RMSEA as suggesting a significant change in model fit (i.e., CFI change ≥ 0.005 and RMSEA change ≥ 0.015 indicate change in fit; Chen 2007). Fit indices specific to bifactor models were also used including Explained Common Variance (ECV > 0.70–0.80 strong general factor), Percentage of Uncontaminated Correlations (PUC > 0.70 indicates low bias and support for unidimensional structure), Coefficient Omega (ω ≥ 0.70 indicates a reliable composite), and Coefficient Omega Hierarchical (ωH > 0.80 suggest the total score can be interpreted as essentially unidimensional; Reise et al. 2010).

Sequential tests of measurement invariance were then conducted to determine the language equivalence between the English/Spanish CGSQ‐SF11 forms for the optimal factor solution based on a significant change in model fit (i.e., configural, metric, scalar; Kline 2023). Configural invariance was examined using a multi‐group CFA fitted for each language form of the CGSQ‐SF11 to determine whether the factor structure is similar across survey versions. Metric invariance was examined by constraining the factor loadings to be equal across groups in the configural model to determine whether items loaded onto the latent factor differently between language forms. Scalar invariance was then assessed by constraining factor loadings and response thresholds (i.e., intercepts) to be equal across groups to determine whether item anchors behaved differently across language versions. Lastly, structural regression models were used to establish the convergent validity of the scale through expected associations between youth internalizing and externalizing problem severity and higher factor scores on the CGSQ‐11. The outcome in these models was a latent variable representing the CGSQ‐SF11, with model specification based on CFA results, and observed variables representing internalizing and externalizing problems t‐scores were the regressors (i.e., independent variables). The amount of variance explained in the latent CGSQ‐SF11 outcome by youth mental health problem severity was also estimated (*r* ^2^).

## 3 Results

Families who completed the Spanish survey significantly differed in several demographic characteristics. Caregivers who completed the Spanish survey were significantly (*p* < 0.001) more likely to identify as female (70.8%), were more likely to be first generation immigrants (65.3%), had lower rates of post‐high school education (56.8%), and lower subjective socioeconomic status (*M* = 6.13, SD = 1.71) relative to those who chose the English survey. Within the present sample, caregivers reported clinically elevated levels of youth internalizing (*M* = 64.3, SD = 13.7) and borderline externalizing problems (*n* = 511; *M* = 62.0, SD = 12.8). Caregivers who completed the Spanish survey also reported significantly lower youth externalizing and internalizing problem severity in the Spanish versus the English survey (*p* < 0.001). See Table 1 for sample characteristics. Bivariate Kendall's Tau correlation between youth internalizing and externalizing *t* scores suggests that there was a moderate positive association between problem types (overall *τ* = 0.52; English *τ* = 0.56; Spanish *τ* = 0.47; *p* < 0.001). In the overall sample, caregivers, on average, reported moderate levels of global strain (*M* = 2.44, SD = 0.97, range = 1–5) based on the possible range of responses on the scale. Objective strain was the highest, followed by subjective internalized and subjective externalized. Caregivers who completed the English CGSQ‐SF11 generally reported higher levels of strain on individual items relative to those who completed the Spanish form (see Table 2). Bivariate Kendall's Tau correlations between CGSQ‐SF11 subscales were positive and of a moderate magnitude within the overall sample (*N* = 511), ranging from 0.42 to 0.57 (*p* < 0.001). Bivariate correlations between CGSQ‐SF11 subscales were also positive and of moderate magnitude within the English (*n* = 275; *τ* = 0.42–0.60) and Spanish versions of the measure (*n* = 236; *τ* = 0.38–0.53; *p* < 0.001).

**TABLE 2:** Summary statistics for the Caregiver Strain Questionnaire‐Short Form 11 items and scales by language form.

Items/scales	Total (*N* = 511)	English (*n* = 275; 53.8%)	Spanish (*n* = 236; 46.2%)	*p*
	*M* (SD)	*M* (SD)	*M* (SD)	
**Objective strain (total *α* = 0.88; English = 0.88; Spanish = 0.86)**	**2.40 (1.10)**	**2.72 (1.13)**	**2.02 (0.95)**	**< 0.001**
(1) Do your child's problems interrupt your personal time?	2.62 (1.25)	2.96 (1.23)	2.22 (1.16)	< 0.001
(2) Do you miss work or neglect other duties because of your child's problems?	2.39 (1.28)	2.72 (1.31)	2.02 (1.13)	< 0.001
(3) Do your child's problems place a financial strain on your family?	2.23 (1.30)	2.60 (1.38)	1.81 (1.05)	< 0.001
(4) Is there disruption or upset of relationships within the family due to your child's problems?	2.36 (1.32)	2.63 (1.36)	2.04 (1.19)	< 0.001
**Subjective internalized strain (total *α* = 0.86; English = 0.86; Spanish = 0.85)**	**2.87 (1.07)**	**3.08 (1.06)**	**2.63 (1.03)**	**< 0.001**
(5) How sad or unhappy do you feel as a result of your child's problems?	2.76 (1.28)	3.04 (1.26)	2.42 (1.22)	< 0.001
(6) How worried do you feel about your child's future?	3.35 (1.31)	3.48 (1.27)	3.20 (1.34)	0.02
(7) How tired or strained do you feel as a result of your child's problems?	2.79 (1.26)	2.97 (1.26)	2.58 (1.23)	< 0.001
(8) In general, how much of a toll do your child's problems take on your family?	2.59 (1.24)	2.82 (1.26)	2.33 (1.17)	< 0.001
**Subjective externalized strain (total *α* = 0.88; English = 0.89; Spanish = 0.81)**	**1.92 (1.11)**	**2.22 (1.23)**	**1.57 (0.82)**	**< 0.001**
(9) How embarrassed do you feel about your child's problems?	2.03 (1.30)	2.37 (1.41)	1.64 (1.02)	< 0.001
(10) How angry do you feel toward your child?	1.99 (1.26)	2.25 (1.37)	1.68 (1.03)	< 0.001
(11) How resentful do you feel toward your child?	1.74 (1.16)	2.05 (1.29)	1.38 (0.84)	< 0.001
**Global strain (total *α* = 0.93; English = 0.93; Spanish = 0.91)**	**2.44 (0.97)**	**2.72 (1.00)**	**2.12 (0.81)**	**< 0.001**

*Note:* *p*‐values represent Kruskal‐Wallis rank sum test examining mean differences for all scales/items between language versions. On average, CGSQ‐SF11 scores on each scale/item were higher in the English relative to the Spanish version. Signals information relating to the global and subscales is provided in bold.

### 3.1 Factor Structure

The unidimensional global factor model (*N* = 511) had a poor fit on the RMSEA, bordered the recommended cut for SRMR, and was in the recommended range for CFI and TLI (see Table 3). Factor loadings for the global scale were high, ranging from 0.64–0.86, which may have contributed to inflation of the RMSEA value (Kline 2023). See Figure 1 for factor loadings for the global factor model on the overall dataset. However, all fit indices for the three‐factor model (*N* = 511) were in the recommended range (i.e., RMSEA, SRMR, CFI, TLI), which may reflect improvements in fit from a reduction in residuals associated with clustering related items (Brown 2015). There were significant decreases in RMSEA (0.074) and CFI (0.011) suggesting that the three‐factor model fit the data significantly better than the unidimensional global factor solution. Factor loadings for the three‐factor model ranged from 0.69 to 0.92 (See Figure S1). Standardized covariances (i.e., correlations) between factors were high, with subjective internalized (0.85) and subjective externalized (0.85) scales having high overlap with objective strain, and a large correlation between subjective internalized and subjective externalized scales (0.72; Brown 2015).

**TABLE 3:** Fit statistics for the Caregiver Strain Questionnaire‐Short Form 11 models.

Models	*N/n*	*X* ^2^	Df	*p*	Root mean square error of approximation [90% CI]	Standardized root mean square residual	Comparative fit index	Tucker‐Lewis index
Measurement								
Unidimensional^a^	511	441.53	44	< 0.001	0.133 [0.122–0.145]	0.089	0.986	0.983
Three‐factor^b^	511	114.73	41	< 0.001	0.059 [0.047–0.072]	0.048	0.997	0.997
Bifactor^c^	511	32.86	33	< 0.001	0.000 [0.000–0.032]	0.028	1.00	1.00
Language invariance^a^								
Configural	511	462.33	88	< 0.001	0.129 [0.118–0.141]	0.094	0.985	0.981
Metric	511	511.68	99	< 0.001	0.128 [0.117–0.139]	0.100	0.983	0.981
Scaler	511	486.55	121	< 0.001	0.109 [0.099–0.119]	0.094	0.985	0.986
Structural regression^a^								
Overall	511	448.22	64	< 0.001	0.108 [0.099–0.118]	0.076	0.988	0.985
English	275	250.63	64	< 0.001	0.103 [0.090–0.117]	0.076	0.989	0.987
Spanish	236	233.17	64	< 0.001	0.106 [0.092–0.121]	0.090	0.983	0.980

^a^ All items loaded onto global factor.

^b^ Items loaded onto objective, subjective internalized, and subjective externalized strain.

^c^ Items loaded onto global factor and objective, subjective internalized or subjective externalized strain factors simultaneously.

**FIGURE 1:**
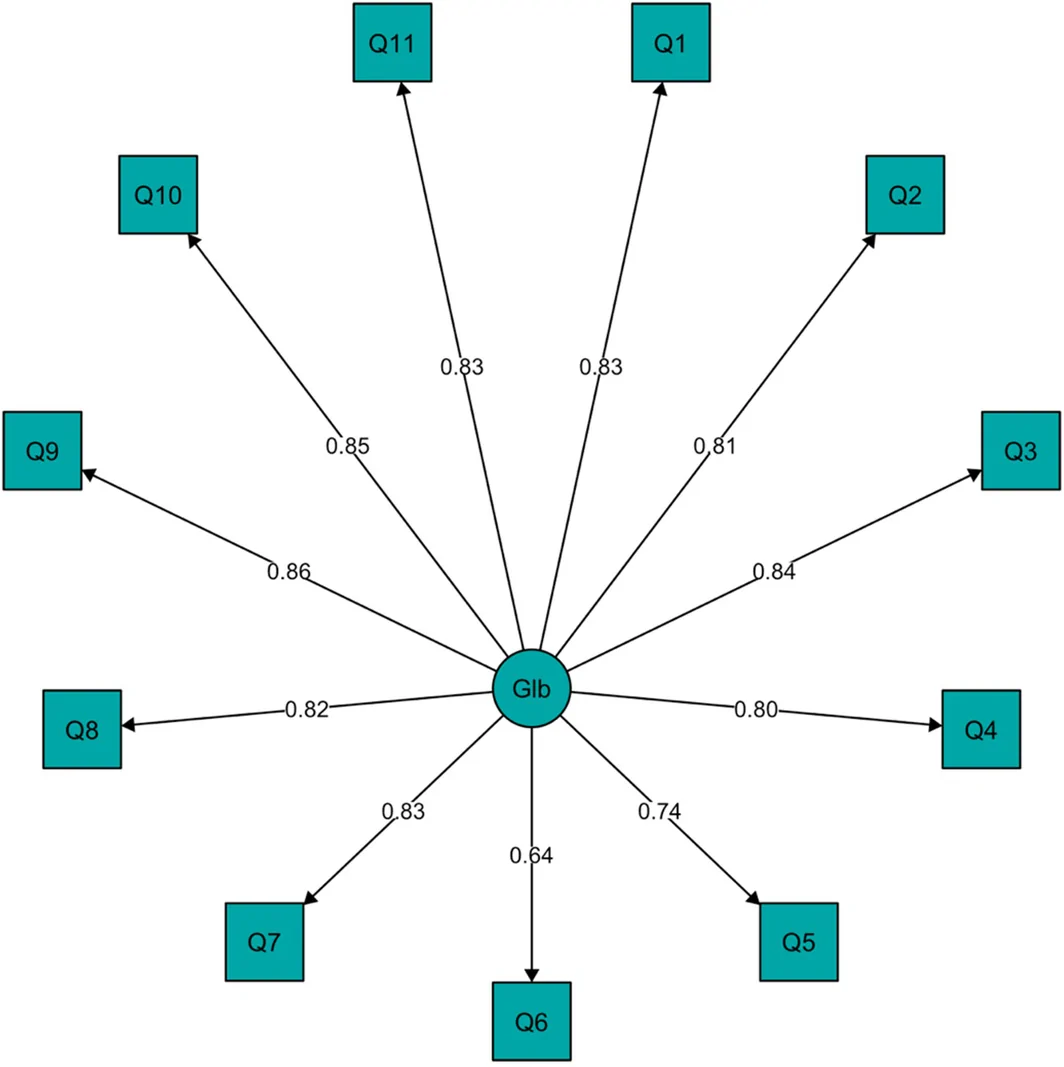
CGSQ‐SF11 global scale factor loadings in the overall sample (*N* = 511). Glb, global scale.

A bifactor model (*N* = 511) was then estimated with items simultaneously loading directly onto a global scale and three‐subscales but resulted in a Heywood case with negative variance. Item two was identified as having a negative residual variance (part of the objective strain subscale); thus, the residual for this item was constrained to zero, which resulted in model convergence. The estimated model had fit indices within the recommended range (i.e., RMSEA, SRMR, CFI, TLI; see Table 3) but only resulted in a significant decrease in RMSEA (0.059) but not CFI (0.003). Factor loadings for the bifactor model ranged from 0.48 to 0.87 for the global factor and −0.07 to 0.73 for the three‐subscales (see Table S1). After accounting for the global factor, items in the objective strain factor largely had weak factor loadings (< 0.150) with item two being the exception (0.582), and item four having a small negative loading (−0.070). The bifactor fit index ECV suggest that 76.3% of the common variance between items was explained by the global factor. PUC suggest that 72.7% of the item correlations reflect only the global factor and were uncontaminated by the subfactors. The *ω* coefficient was high (0.956) and suggest the total score reflected common variance rather than measurement error. Lastly, 87.2% of the reliable variability was attributed to the global factor (*ωH*). Inconsistent with our hypotheses, these findings suggest that the measure is best represented by a unidimensional global factor as the additional three‐factors only accounted for a limited amount of unique variance.

### 3.2 Language‐Based Measurement Invariance

Measurement invariance test across language forms was conducted with the unidimensional factor model. First, a configural multi‐group CFA model was fitted by language version (English *n* = 275; Spanish *n* = 236) and found that the global factor fit the data similarly to the analysis conducted on the overall sample according to CFI (≥ 0.01) and RMSEA (≥ 0.015) changes that did not exceed the significant range. See Figure S2 for factor loadings by language version. A metric model was then fitted by constraining factor loading to be equal across the English and Spanish language forms. Metric invariance across languages was substantiated through CFI (≥ 0.01) and RMSEA (≥ 0.015) changes that did not exceed the significant range. A scalar model was then fitted with response thresholds constrained to be equal across models with a significant change in RMSEA (0.019) suggesting a lack of scalar invariance. These findings suggest that participants' responses across language versions loaded similarly onto the same global factor, but response anchors may behave differently between forms.

### 3.3 Convergent Validity

The global caregiver strain scale had excellent internal consistency within the overall sample and by language form (*α* = 0.93; English = 0.93; Spanish = 0.91). Bivariate Kendall's Tau correlations between the CGSQ‐SF11 global scale and youth externalizing (*τ* = 0.49; *p* < 0.001) and internalizing problem *t* scores (*τ* = 0.38; *p* < 0.001) suggested moderate positive associations within the overall sample (*N* = 511). When examined separately by form, bivariate Kendall's Tau correlations between the CGSQ‐SF11 global scale and youth externalizing and internalizing problem *t* scores also had moderate positive associations, with an increase in youth problem severity being associated with higher global strain within English (*n* = 275; youth externalizing *τ* = 0.47 and internalizing problems *τ* = 0.42; *p* < 0.001) and Spanish versions of the measure (*n* = 236; youth externalizing *τ* = 0.47 and internalizing problems *τ* = 0.32; *p* < 0.001).

Structural regression models were then fitted on the overall sample and by language version with a unidimensional latent outcome representing global CGSQ‐SF11 strain scores and observed CBCL youth internalizing and externalizing *t* scores as the simultaneous regressors (i.e., predictors). The overall sample (*N* = 511) structural regression model had acceptable fit according to SRMR, CFI, and TLI, but had poor fit according to RMSEA (see Table 3); however, collectively global fit was deemed acceptable. Within the overall sample, youth internalizing and externalizing problem *t* scores were positively and uniquely associated with global strain and explained 46.8% of the variance in the latent outcome. However, youth externalizing problems had a large association with global caregiver strain (*β* = 0.55) relative to a small association for internalizing problems (*β* = 0.17) when controlling for shared variance (i.e., general psychopathology). The English sample (*n* = 275) model had acceptable fit according to SRMR, CFI, and TLI, but had poor fit according to RMSEA (see Table 3). Within the English sample, youth internalizing and externalizing problem *t* scores were also positively associated with global strain, explained 46.2% of the variance in the latent outcome, and externalizing problems had a moderate effect (*β* = 0.47) relative to a small effect (*β* = 0.26) for internalizing problems. The Spanish sample (*n* = 236) model had acceptable fit according to CFI and TLI, marginally poor fit on SRMR, and poor fit according to RMSEA (see Table 3). Within the Spanish sample, the model explained 43.6% of the variance in the latent outcome with youth externalizing problem *t* scores being positively associated with global strain (*β* = 0.62) whereas internalizing problems was not significantly associated with the outcome. See Table 4 for structural regression model estimates.

**TABLE 4:** Structural regression of youth mental health problems association with the Caregiver Strain Questionnaire‐Short Form 11 global scale.

Variables	*b*	*β*	SE	*p*	*R* ^2^
*Overall (N = 511)*					
Global strain (latent outcome)					0.468
Internalizing	0.010	0.171	0.003	< 0.001	
Externalizing	0.036	0.553	0.003	< 0.001	
*English (n = 275)*					
Global strain (latent outcome)					0.462
Internalizing	0.016	0.258	0.004	< 0.001	
Externalizing	0.030	0.469	0.004	< 0.001	
*Spanish (n = 236)*					
Global strain (latent outcome)					0.436
Internalizing	0.004	0.059	0.004	0.337	
Externalizing	0.040	0.620	0.003	< 0.001	

Abbreviations: *β*, standardized estimate; *b*, unstandardized estimate; SE, standard error.

## 4 Discussion

The present study extends prior research by translating the CGSQ‐SF11 into Spanish and conducting a psychometric evaluation of the measure within a sample of Latinx caregivers. Overall, caregivers reported moderate levels of caregiver strain, and caregivers who completed the Spanish survey largely reported significantly lower youth mental health problem severity and caregiver strain relative to those who completed the English survey. Findings from the CFA suggest that the three‐factor structure of the CGSQ‐SF11 fit the data better than a unidimensional global strain factor. However, a bifactor model uncovered instability in the objective strain scale within the three‐factor model after accounting for the global factor, and bifactor fit indices suggest that the CGSQ‐11 is best represented by a single factor despite having relatively poorer fit when compared to the three‐factor model. Thus, the CGSQ‐SF11 may be best represented as a unidimensional global score that had excellent internal consistency and displayed metric invariance across English and Spanish forms. While prior research has identified the three‐factor solution as the optimal structure for representing the English CGSQ, differences in our conclusions may stem from the lack of bifactor analyses in prior research needed to determine whether a multidimensional solution is justified beyond the unidimensional scale (Brennan et al. 2021; Domenech Rodríguez et al. 2024). This finding has important research implications, as a brief scale like the CGSQ‐SF11 can facilitate research on caregiver strain across Spanish and English‐speaking Latinx families to expand our understanding of this perceptual factor impacting health disparities within this group.

Descriptive analysis found lower mean CGSQ‐SF11 scores in the Spanish survey relative to the English version. Our results suggest that the Spanish survey group, despite recognizing the presence of emotional or behavioral problems in their children, reported lower severity of mental health problem symptoms in their youth and reported lower associated caregiver strain on CGSQ‐SF11 scales and individual items. As participants who completed the Spanish survey were more likely to be first generation immigrants relative to those who chose the English version, it is possible that observed differences in youth symptomology and caregiver strain may represent potential protective effects of greater alignment with cultural values that may moderate the impact of contextual stressors on Latinx caregivers (e.g., personalism, familism, fatalism, respect; Cardoso and Thompson 2010). As survey panel research is a nonprobability sampling approach, differences in youth problem severity and caregiver strain between the English and Spanish versions may also represent selection biases related to who chooses to or was able to participate in the study, which may contribute to group‐based differences (e.g., potential overrepresentation of higher socioeconomic status and more acculturated Latinx caregivers in the English survey; Vázquez, Chou, et al. 2021). It is also possible that differences in responses on CGSQ‐SF11 items and scales between the English and Spanish forms may be due to perceptual differences between groups that influenced self‐reported caregiver strain, and thus further research is needed to determine whether these differences are mitigated through more objective measures of caregiver strain. Future research may examine in detail the types of varying demographic characteristics and experiences that may contribute to lower perceived severity among caregivers and potential differences between subjective and objective measures.

While caregiver responses on the CGSQ‐SF11 differed between languages at the mean level, a unidimensional global factor representing the scale was confirmed on a sample combining responses from both language versions (Domenech Rodríguez et al. 2024). Constraining factor loadings to be equal did not significantly change the model fit, which suggests that the strength of relations between items and latent factors did not significantly differ across language forms. These findings indicate that the CGSQ‐SF11 demonstrates metric invariance across language versions. As a result, significantly lower CGSQ‐SF11 scale scores in the Spanish survey appear to be in part a function of lower youth problem severity within this sample rather than measurement issues related to the instrument. However, we did document some differential item functioning as scalar invariance was not achieved, which suggests that response anchors may behave differently between forms. This finding is consistent with prior research evaluating measurement invariances of psychological measures between culturally and linguistically diverse groups, which suggests that metric invariance is relatively commonly achieved but scalar invariance is frequently not supported as groups often vary on norms, social desirability, linguistic connotations, and social meaning of the construct being assessed (Davidov et al. 2014; Rutkowski and Svetina 2014; Zercher et al. 2015).

Our findings suggest that the English and Spanish versions of the CGSQ‐SF11 appear to be reliable measures of caregiver strain with excellent internal consistency across language forms. This finding further supports the use of the CGSQ‐SF11 English and Spanish forms with Latinx caregivers. Consistent with prior research, the present study examined associations with youth mental health problem severity as an indicator of convergent validity for the CGSQ‐SF11 as caregiver strain theoretically emerges in response to youth emotional and/or behavioral problems (Brannan et al. 1997, 2021). Findings from the present study support the convergent validity of the CGSQ‐SF11 through moderate positive bivariate correlations between the global scale and indicators of youth mental health problem severity within the overall sample and within each language form (Domenech Rodríguez et al. 2024). Structural regression models fitted on the overall sample and by language form found that, when modeled simultaneously as predictors, CBCL youth internalizing and externalizing *t* scores explained between 43.6% and 46.8% of the variance in latent global strain scores. Findings from the multivariate structural regression models suggest that increases in youth externalizing problem severity were consistently and uniquely associated with a larger increase in the latent global strain outcome relative to internalizing problems across language versions. Further, increases in youth internalizing and externalizing problems severity were associated with greater latent global strain for caregivers within the overall and English language samples. Only externalizing problems were uniquely associated with latent global strain, when controlling for internalizing problems, within the Spanish language sample. This finding may reflect lower acculturation and greater alignment with Latinx cultural expectation that youth should be well‐behaved and listen to their caregivers, which may contribute to externalizing problems being particularly stigmatizing and stressful for caregivers within the Spanish language sample (Domenech Rodríguez et al. 2024; Vázquez, Culianos, Gudiño, et al. 2024; Vázquez, Domenech Rodríguez, Navarro Flores, et al. 2024). Despite these promising findings regarding reliability and convergent validity, further research is needed to determine whether the CGSQ‐SF11 provides incremental utility in predicting psychological outcomes and help‐seeking behaviors among Latinx caregivers relative to measures that assess parenting stress more broadly (e.g., Parenting Stress Index–Short Form; Reitman et al. 2002).

### 4.1 Implications

Our findings suggest that researchers can use data that combines responses from the English and Spanish versions of the CGSQ‐SF11. However, as scalar invariance was not achieved, some differences in CGSQ‐SF11 scores between language versions may be observed due to item anchors functioning differently between the English and Spanish forms. Latinx families have experienced notable stress over the past decade associated with a more uncertain sociopolitical climate that contributes to mental health problems (Held et al. 2022). The availability of psychometrically evaluated tools is essential to accurately detect distress specifically related to youth emotional or behavioral problems, particularly in periods of elevated general stress.

While brief measures of broader parenting stress such as the PSI‐SF have been psychometrically evaluated among Latinx families (Hong and Rios 2024), this instrument may not be the most conceptually appropriate or efficient measure for assessing strain specifically resulting from caring for a youth with emotional and/or behavioral problems (Brannan et al. 1997). The CGSQ‐SF11 has several advantages over the PSI‐SF for research and clinical practice focused on supporting caregivers experiencing strain resulting from youth emotional and/or behavioral problems. Although prior research in a German sample found that the CGSQ‐SF11 global score and PSI‐SF were highly correlated (*r* = 0.75), the CGSQ‐SF11 provides greater conceptual specificity by assessing caregiver strain specifically associated with children's emotional and/or behavioral problems relative to the PSI‐SF that assesses broader parenting stress (Göldel and Warschburger 2024). This distinction is important when the goal of research or clinical interventions is to understand and address caregivers' responses to their youth's mental health problems rather than stress resulting from parenting more generally (Brannan et al. 1997; Reitman et al. 2002). Moreover, the CGSQ‐SF11 includes items that map onto theoretically derived domains of caregiver strain that are not explicitly differentiated within the PSI‐SF (i.e., objective strain and subjective internalized and externalized strain; Brennan et al. 2021; Reitman et al. 2002). Thus, the CGSQ‐SF11 may capture distinct dimensions of caregivers' experiences that are particularly relevant to understanding the consequences of youth emotional and behavioral problems. Finally, although both measures were developed as brief instruments, the 36‐item PSI‐SF is substantially longer than the 11‐item CGSQ‐SF11 (Brennan et al. 2021; Reitman et al. 2002). Thus, the brevity of the CGSQ‐SF11 may reduce barriers to the inclusion of caregiver strain measures in research and clinical practice by reducing respondent burden and increases feasibility of repeated administrations. Nonetheless, as previously noted, future research is required to evaluate the incremental predictive utility of each measure.

An important direction for future research is to determine the potential utility of the CGSQ‐SF11 in a preventative context. Because elevated caregiver strain may inform parental recognition of youth mental health symptoms, their severity, and utilization of services (Brennan et al. 2021; Vázquez, Chou, et al. 2021; Domenech Rodríguez et al. 2024), the CGSQ‐SF11 may facilitate the identification of parents who need additional support in recognizing and seeking care for their youth. Research supports that recognition of youth's mental health needs will most often prompt care‐seeking, even when parents have stigmatized beliefs about mental health and related services (Dixon De Silva et al. 2020). Detection of elevated caregiver strain in parents can be a step toward identifying individuals with whom providers should have more in‐depth conversations about the sources and extent of that strain, including externalizing and internalizing symptoms within their youth. These conversations can, in turn, facilitate effective treatment seeking. Future research could also increase the clinical utility of the CGSQ‐SF11 by using Area Under the Curve analyses to identify threshold scores associated with help‐seeking for youth and risk for developing clinically elevated mental health problems among Latinx caregivers of youths.

### 4.2 Limitations

Several limitations should be noted when interpreting the findings of the present study. Recruitment occurred during a period of elevated caregiver strain due to Coronavirus Pandemic related stressors, which may have inflated ratings of both youth and caregiver experiences (Vázquez, Navarro Flores, et al. 2021). Research is needed to confirm our findings with a post pandemic sample. Future research should also replicate our findings using a pairwise comparison of Latinx families matched on demographic characteristics to determine whether this explains differences in caregiver strain reporting between English‐Spanish versions of the CGSQ‐SF11. Future studies should use a longitudinal design and a sample with greater youth mental health risk variation to obtain more nuanced insights into our findings. As participants in the present study identified predominantly as female, research is needed to confirm the factor structure of the CGSQ‐SF11 within a sample of Latinx men to determine the generalizability of our findings. This is important as there may be gender‐based differences in caregiver strain resulting from cultural expectations regarding who is responsible for child rearing, which affects degree of exposure to caring for a child with emotional and/or behavioral problems (Vázquez, Navarro Flores, et al. 2021).

The present study recruited participants using a survey panel approach, which includes potential introduction of selection biases associated with the composition of these panels and strategies for protecting against threats to data integrity. Online survey panels may have overrepresentation of higher socioeconomic status and more acculturated Latinx caregivers, which may impact the generalizability of our findings to the broader Latinx population in the U.S. (Vázquez, Navarro Flores, et al. 2021). Only data from participants that provided high quality and complete responses on the survey were used to protect against malicious responders and bots who provided inattentive, incomplete, and/or nonsensical responses to receive survey rewards (Lowry et al. 2016; Kennedy et al. 2020). It is possible that participants who were uncomfortable with disclosing information about their caregiver strain or youth's mental health problems may have been more likely to discontinue participation. The use of attention‐check to validate responses and protect data integrity may have also led to under‐representation of caregivers with lower literacy levels who may have been more likely to fail consistency checks. However, we were unable to analyze differences between respondents with complete and incomplete data or use imputation methods as QuestionPro was only contracted to provide complete and high‐quality responses at the end of the recruitment phase. Future studies should examine the structure of the CGSQ‐SF11 using sampling strategies that allow for the examination of mechanisms of missingness and imputation of missing data. Future research should also evaluate the CGSQ‐SF11 in more diverse Latinx samples. Despite these limitations, this research establishes support for a tool that can measure caregiver strain in both English and Spanish within Latinx communities.

## 5 Conclusion

The present study translated the CGSQ‐SF11 into Spanish and conducted a psychometric evaluation within a sample of Latinx caregivers. Our analyses suggest that while the three‐factor structure of the CGSQ‐SF11 fit the data better than a global strain factor, findings from a bifactor model largely supported a unidimensional factor as the optimal structure. The unidimensional factor structure for the CGSQ‐SF11 global scale did not significantly differ across language forms, demonstrating metric invariance. The availability of an English/Spanish CGSQ‐SF11 provides a brief instrument that assesses experiences consistent with theories of caregiver strain, which can facilitate the identification of related correlates in health disparities research and can be used in clinical practice to identify caregivers in need of additional support to improve caregiver and youth outcomes.

## Funding

This research was funded by the Culture and Mental Health Lab at Utah State University.

## Ethics Statement

The original protocol was approved by the Utah State University—Institutional Review Board (#12087). The University of Tennessee, Knoxville—Institutional Review Board approved analysis of the extant de‐identified data as nonhuman subjects research for the present study. All participants provided informed consent prior to engaging in the study.

## Conflicts of Interest

The authors declare no conflicts of interest.

## Supporting information


**Table S1:** Factor loadings for the bifactor model on overall sample (*N* = 511).
**Figure S1:** The Caregiver Strain Questionnaire‐Short Form 11 three‐factor model in the overall sample (*N* = 511).
**Figure S2:** Factor loadings for the Caregiver Strain Questionnaire‐Short Form 11 global scale by language version.

## Data Availability

All data and analysis code for this study will be available by emailing the corresponding author.
