# Supplementary material for: Establishing the Structural Validity, Language‐Based Performance, and Convergent Validity of the Caregiver Strain Questionnaire‐Short Form 11 Within a Latinx Sample
Source: Fam Process. 2026 Nov 1;65(4):e70213. doi: 10.1111/famp.70213 (PMC13724282; doi:10.1111/famp.70213)
Supplement: Supplementary file 1 [file FAMP-65-0-s001.docx]

**Supplemental Materials**

**Caregiver Strain Questionnaire – Short Form 11**

We are trying to understand how things are for you and your family and how much your child’s problems or difficulties have affected you and your family. For each question below, please check the response that best answers the question for you and your family at the present time.

|  | Not at all  (1) | A little  (2) | Somewhat (3) | Quite a bit  (4) | Very much (5) |
| --- | --- | --- | --- | --- | --- |
| 1) Do your child’s problems interrupt your personal time? |  |  |  |  |  |
| 2) Do you miss work or neglect other duties because of your child’s problems? |  |  |  |  |  |
| 3) Do your child’s problems place a financial strain on your family? |  |  |  |  |  |
| 4) Is there disruption or upset of relationships within the family due to your child’s problems? |  |  |  |  |  |
| 5) How sad or unhappy do you feel as a result of your child’s problems? |  |  |  |  |  |
| 6) How worried do you feel about your child’s future? |  |  |  |  |  |
| 7) How tired or strained do you feel as a result of your child’s problems? |  |  |  |  |  |
| 8) In general, how much of a toll do your child’s problems take on your family? |  |  |  |  |  |
| 9) How embarrassed do you feel about your child’s problems? |  |  |  |  |  |
| 10) How angry do you feel toward your child? |  |  |  |  |  |
| 11) How resentful do you feel toward your child? |  |  |  |  |  |

**Caregiver Strain Questionnaire – Short Form 11**

Estamos tratando de entender cómo son las cosas para usted y su familia, y cuánto los problemas o dificultades de su hijo le han afectado a usted y a su familia. Para cada pregunta a continuación, por favor marque la respuesta que mejor capture la experiencia para usted y su familia actualmente.

|  | Nada  (1) | Un poco  (2) | Algo  (3) | Bastante  (4) | Muchísimo  (5) |
| --- | --- | --- | --- | --- | --- |
| 1) ¿Los problemas de su hijo/a interrumpen su tiempo personal? |  |  |  |  |  |
| 2) ¿Falta al trabajo o descuida otras tareas debido a los problemas de su hijo/a? |  |  |  |  |  |
| 3) ¿Los problemas de su hijo/a representan una carga económica para su familia? |  |  |  |  |  |
| 4) ¿Hay interrupción o alteración de las relaciones dentro de la familia debido a los problemas de su hijo/a? |  |  |  |  |  |
| 5) ¿Qué tan triste o infeliz se siente como resultado de los problemas de su hijo/a? |  |  |  |  |  |
| 6) ¿Qué tan preocupada se siente por el futuro de su hijo/a? |  |  |  |  |  |
| 7) ¿Qué tan cansado o tenso se siente como resultado de los problemas de su hijo/a? |  |  |  |  |  |
| 8) ¿En general, qué consecuencias tienen los problemas de su hijo/a en su familia? |  |  |  |  |  |
| 9) ¿Qué tan avergonzado se siente por los problemas de tu hijo/a? |  |  |  |  |  |
| 10) ¿Qué tan enojado se siente con su hijo/a? |  |  |  |  |  |
| 11) ¿Cuánto resentimiento siente hacia su hijo/a? |  |  |  |  |  |

**Table S1**

*Factor Loadings for the Bifactor Model on Overall Sample (*N *= 511)*

| Items | Objective strain | Internalized strain | Externalized strain | Global strain |
| --- | --- | --- | --- | --- |
| Q1 | .150 | - | - | .840 |
| Q2 | .582 | - | - | .813 |
| Q3 | .077 | - | - | .865 |
| Q4 | -.070 | - | - | .852 |
| Q5 | - | .399 | - | .682 |
| Q6 | - | .731 | - | .482 |
| Q7 | - | .459 | - | .757 |
| Q8 | - | .293 | - | .791 |
| Q9 | - | - | .414 | .799 |
| Q10 | - | - | .548 | .748 |
| Q11 | - | - | .436 | .750 |

**Figure S1**

*The Caregiver Strain Questionnaire-Short Form 11 Three-Factor Model in the Overall Sample (*N *= 511)*

*Note.* OBJ = objective; EXT = subjective externalized; INT = subjective internalized.

**Figure S2**

*Factor Loadings for the Caregiver Strain Questionnaire-Short Form 11 Global Scale by Language Version*

*Note.* Glb = Global scale. English *n* = 275, Spanish *n* = 236.
